# P-301. What Resistant Bacteria Are Lurking Outside Our Hospitals?

**DOI:** 10.1093/ofid/ofae631.504

**Published:** 2025-01-29

**Authors:** Yasna Yusuf, Olubosede Awoyomi, Yongsheng Wang, Alison Bradbury, Briana Episcopia, Marie Abdallah, John Quale

**Affiliations:** New York City Health and Hospitals/Kings County/SUNY Downstate, Brooklyn, New York; NYC health and hospitals/Kings County, Brooklyn, New York; NYC Health + Hospitals/Kings County, Brooklyn, New York; NYC health and hospitals/Kings County, Brooklyn, New York; NYC H+H/ Kings County, Garden City, New York; NYC health and hospitals/Kings County, Brooklyn, New York; New York City Health and Hospital/Kings County, Brooklyn, New York

## Abstract

**Background:**

Contamination of hospital environmental surfaces with resistant Enterobacterales and non-fermenting bacilli can facilitate nosocomial transmission. Little is known regarding the extent of contamination of environmental surfaces immediately outside the hospital setting.

**Methods:**

Cultures of environmental surfaces surrounding hospitals in New York City were incubated at 37^o^C in MacConkey broth containing 4 mg/L ceftazidime and amphotericin B. Two sets of cultures were obtained: Group 1 cultures involved 42 high-touch surfaces within a two-block radius of each hospital that were cultured with rayon-tipped swabs. Group 2 cultures involved 5 surfaces of transportation centers near (n=8) and distant (n=8) from hospitals that were wiped with saline-saturated sterile gauze. Bacteria were identified using 16S ribosomal sequencing; β-lactamases were identified by PCR sequencing.

**Results:**

In Group 1, the 336 swab cultures from surfaces around 8 hospitals yielded 66 cephalosporin-resistant bacteria, including 26 *Brucella* (*Ochrobactrum*) spp. and 12 *Acinetobacter* spp. One isolate *of Brucella* (*Ochrobactrum*) spp. was found to possess the carbapenemase OXA-24. Surfaces around 2 of the 8 hospitals accounted for 73% of the *Brucella* (*Ochrobactrum*) spp.

In Group 2, the 40 gauze cultures of transportation center surfaces near 8 hospitals yielded 38 bacteria. Of the 38 bacteria, 15 were *Brucella* (*Ochrobactrum*) spp. and 8 were *Acinetobacter* spp. In comparison, the 40 gauze cultures of 8 transportation center surfaces distant from hospitals yielded 33 isolates, including only *5 Brucella* (*Ochrobactrum*) spp. (P=.03 compared to cultures near hospitals) and 6 *Acinetobacter* spp. No members of Enterobacterales were recovered in any of the Group 1 or Group 2 cultures.

**Conclusion:**

Cephalosporin-resistant Enterobacterales were not recovered in any environmental cultures around hospitals. Both *Brucella* (*Ochrobactrum*) spp. and *Acinetobacter* spp. were frequently recovered; *Brucella* (*Ochrobactrum*) spp. appeared to be territorial and a reservoir for OXA-type carbapenemases.

**Disclosures:**

**All Authors**: No reported disclosures

